# Author Correction: Palmitoylation of ULK1 by ZDHHC13 plays a crucial role in autophagy

**DOI:** 10.1038/s41467-024-52646-2

**Published:** 2024-10-03

**Authors:** Keisuke Tabata, Kenta Imai, Koki Fukuda, Kentaro Yamamoto, Hayato Kunugi, Toshiharu Fujita, Tatsuya Kaminishi, Christian Tischer, Beate Neumann, Sabine Reither, Fatima Verissimo, Rainer Pepperkok, Tamotsu Yoshimori, Maho Hamasaki

**Affiliations:** 1 Laboratory of Intracellular Membrane Dynamics, Graduate School of Frontier Biosciences, Osaka University, Osaka, Japan; 2 Department of Genetics, Graduate School of Medicine, Osaka University, Osaka, Japan; 3 Integrated Frontier Research for Medical Science Division, Institute for Open and Transdisciplinary Research Initiatives (OTRI), Osaka University, Osaka, Japan; 4 Advanced Light Microscopy Facility, EMBL, Heidelberg, Germany; 5 Cell Biology and Biophysics Unit, EMBL, Heidelberg, Germany

**Keywords:** Autophagy, Post-translational modifications, Autophagosomes

Correction to: *Nature Communications* 10.1038/s41467-024-51402-w, published online 21 August 2024

In the Acknowledgements section of this article, the grant number was incorrectly given as ‘JSPS KAKENHI JP26251020’ and should have been ‘JSPS KAKENHI JP26251020 22H04982’. The original article has been corrected.

