# Supplementary material for: Palmitoylation of ULK1 by ZDHHC13 plays a crucial role in autophagy
Source: Nat Commun. 2024 Aug 21;15:7194. doi: 10.1038/s41467-024-51402-w (PMC11339336; doi:10.1038/s41467-024-51402-w)
Supplement: Supplementary file 1 — Supplementary Information [file 41467_2024_51402_MOESM1_ESM.pdf]

Supplemental Information

Palmitoylation of ULK1 by ZDHHC13 plays a crucial role in autophagy

Keisuke Tabata[1,2], Kenta Imai[1,2], Koki Fukuda[1,2], Kentaro Yamamoto[1,2], Hayato Kunugi[1,2], Toshiharu Fujita[1,2], Tatsuya Kaminishi[2,3], Christian Tischer[4], Beate Neumann[4], Sabine Reither[4], Fatima Verissimo[4], Rainer Pepperkok[4,5], Tamotsu Yoshimori[1,2,3*] and Maho Hamasaki[1,2*]

[1] Laboratory of Intracellular Membrane Dynamics, Graduate School of Frontier Biosciences, Osaka University, Osaka, Japan
[2] Department of Genetics, Graduate School of Medicine, Osaka University, Osaka, Japan
[3] Integrated Frontier Research for Medical Science Division, Institute for Open and Transdisciplinary Research Initiatives (OTRI), Osaka University, Osaka, Japan
[4] Advanced Light Microscopy Facility, EMBL, Heidelberg, Germany
[5] Cell Biology and Biophysics Unit, EMBL, Heidelberg, Germany

[*] Correspondence: hamasaki@fbs.osaka-u.ac.jp; tamyoshi@fbs.osaka-u.ac.jp

This PDF file includes:
Supplementary Fig. 1-4
Supplementary Table 1
Supplementary References

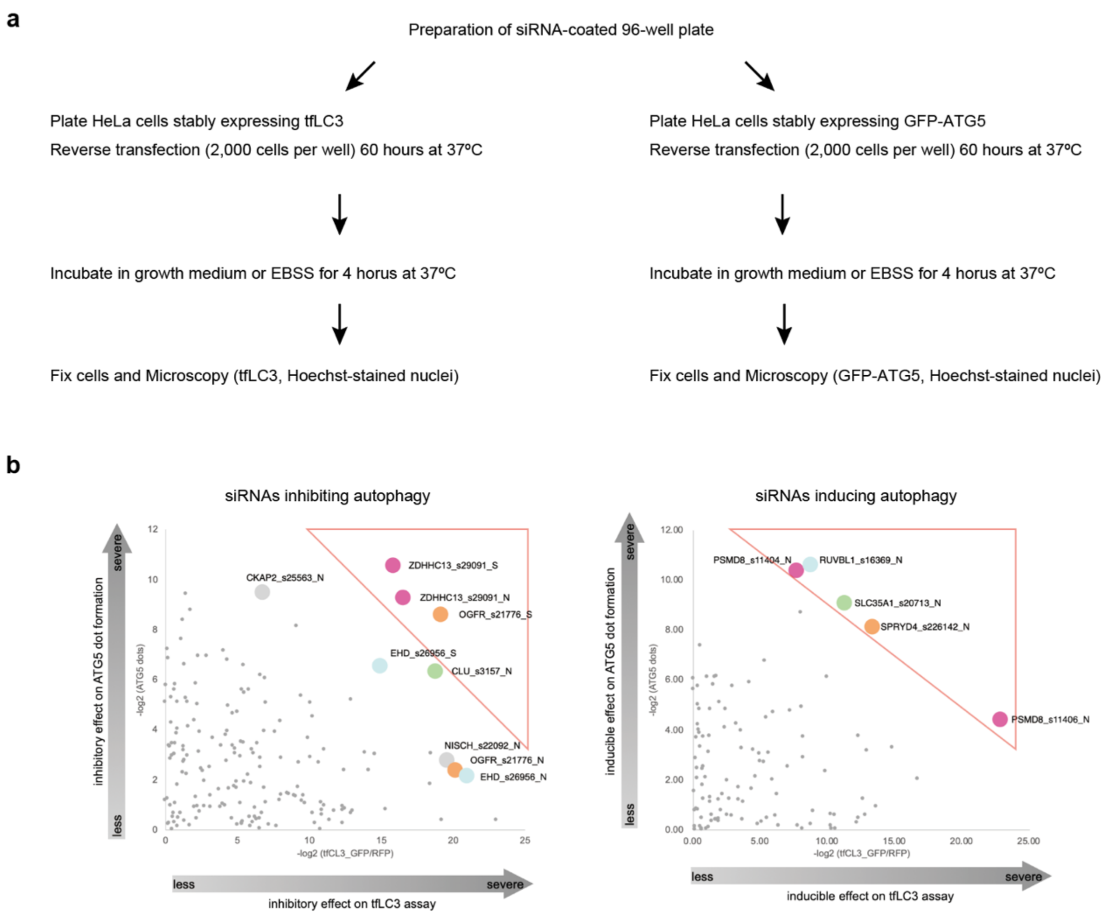

Supplementary Fig. 1

Identification of ZDHHC13 as an autophagy regulating factor from siRNA screening

a Overview of siRNA screening. HeLa cells stably expressing tfLC3 or GFP-ATG5 were plated in 96-well plates where siRNAs and transfection regents were coated. These experiments were independently repeated three times. Images were automatically acquired by using an Olympus scan^R fluorescence microscope. **b** Summary of screening results. To investigate the effect on autophagy flux in tfLC3 assay, signal intensities of RFP and GFP were measured and calculated as a ratio of total GFP signal / total RFP signal. To examine the effect on autophagy induction or autophagosome formation, GFP-ATG5 dot formation per cells were analyzed. *P* values from these experiments were shown as -log2. In left graph, siRNA and target genes were collected by definition as following; GFP/RFP ratio was more than 1.0, GFP-ATG5 dots per cell were less than that in siControl-treated cells. In right graph, siRNA and target genes

were collected by definition as following; GFP/RFP ratio was less than 1.0, GFP-ATG5 dots per cell were more than that in siControl-treated cells.

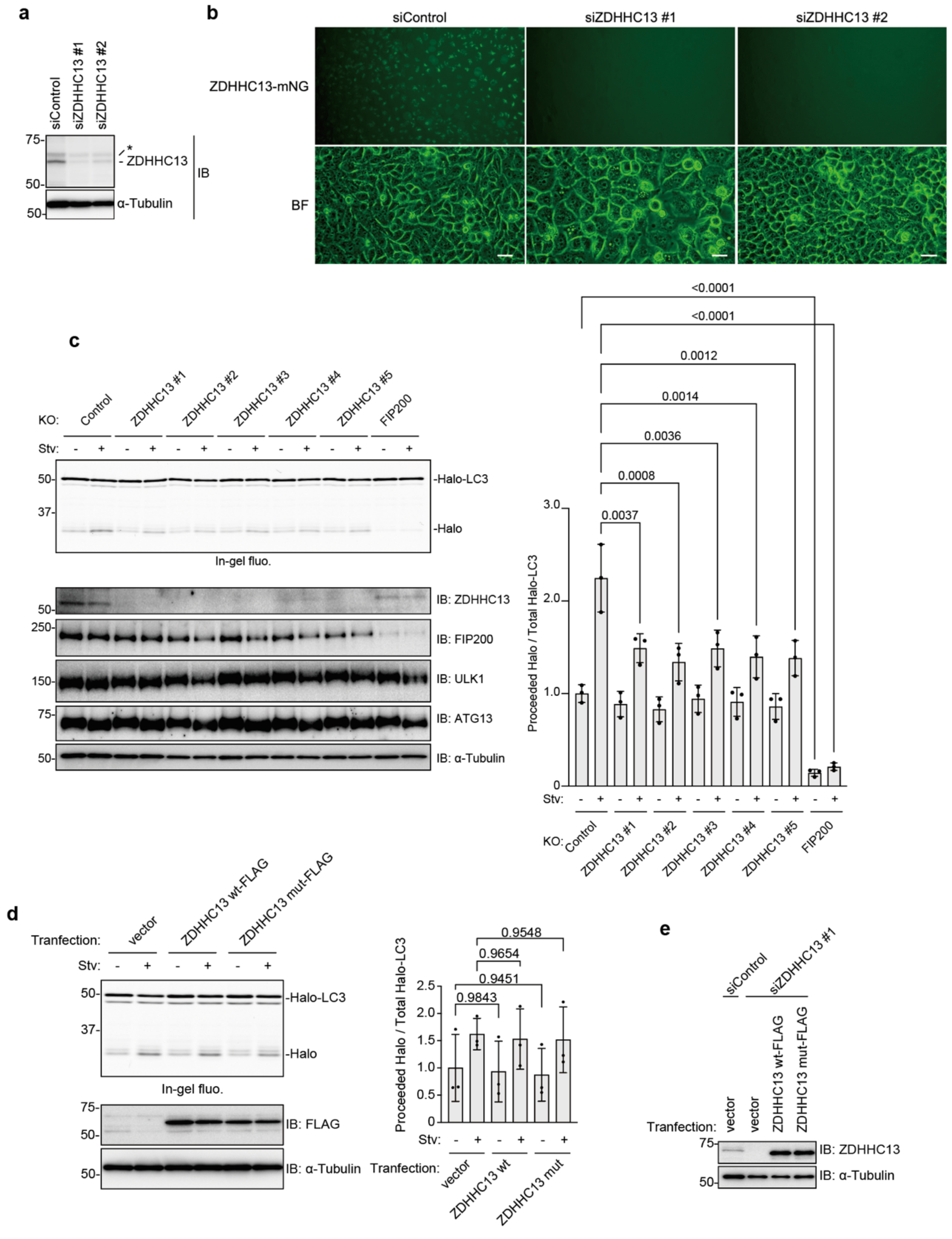

Supplementary Fig. 2

ZDHHC13 knockdown and knockout impaired autophagy flux.

a Cells were transfected with siControl, siZDHHC13 #1 or siZDHHC13 #2 for 2 days. Cell lysate from knockdown cells were analyzed by immunoblotting with anti-ZDHHC13 or anti-α-tubulin antibody. Molecular weights are labeled on the left of each blot. Non-specific bands are indicated as * **b** HeLa cells stably expressing ZDHHC13-mNG were treated with siControl or siZDHHC13 for 2 days. Top panels show mNG signals. Bottom images were acquired by bright field (BF). Scale bars show 50 μm. **c** HeLa cells stably expressing Halo-LC3 were infected with lentivirus harboring sgRNA against control or ZDHHC13. After 5 days, the cells were subjected to pulse-chase reporter processing assay. Cells were pulse-labeled for 20 min with TMR-conjugated ligands and incubated in growth medium or starvation medium for 6 h. Representative images from in-gel fluorescence and Immunoblotting are show in left. The graph is represented as mean ± SD from three experiments. Significance was calculated by one way-ANOVA. **d** Effect of ZDHHC13 overexpression on autophagy flux. HeLa cells expressing Halo-LC3 were transfected either with vector, ZDHHC13 wt or mutant. Autophagy flux was analyzed as described in c. Representative images from in-gel fluorescence and Immunoblotting are show in left. The graph is represented as mean ± SD from three experiments. Significance was calculated by one way-ANOVA. **e** Expressions were analyzed by immunoblotting. Related to Fig.1c.

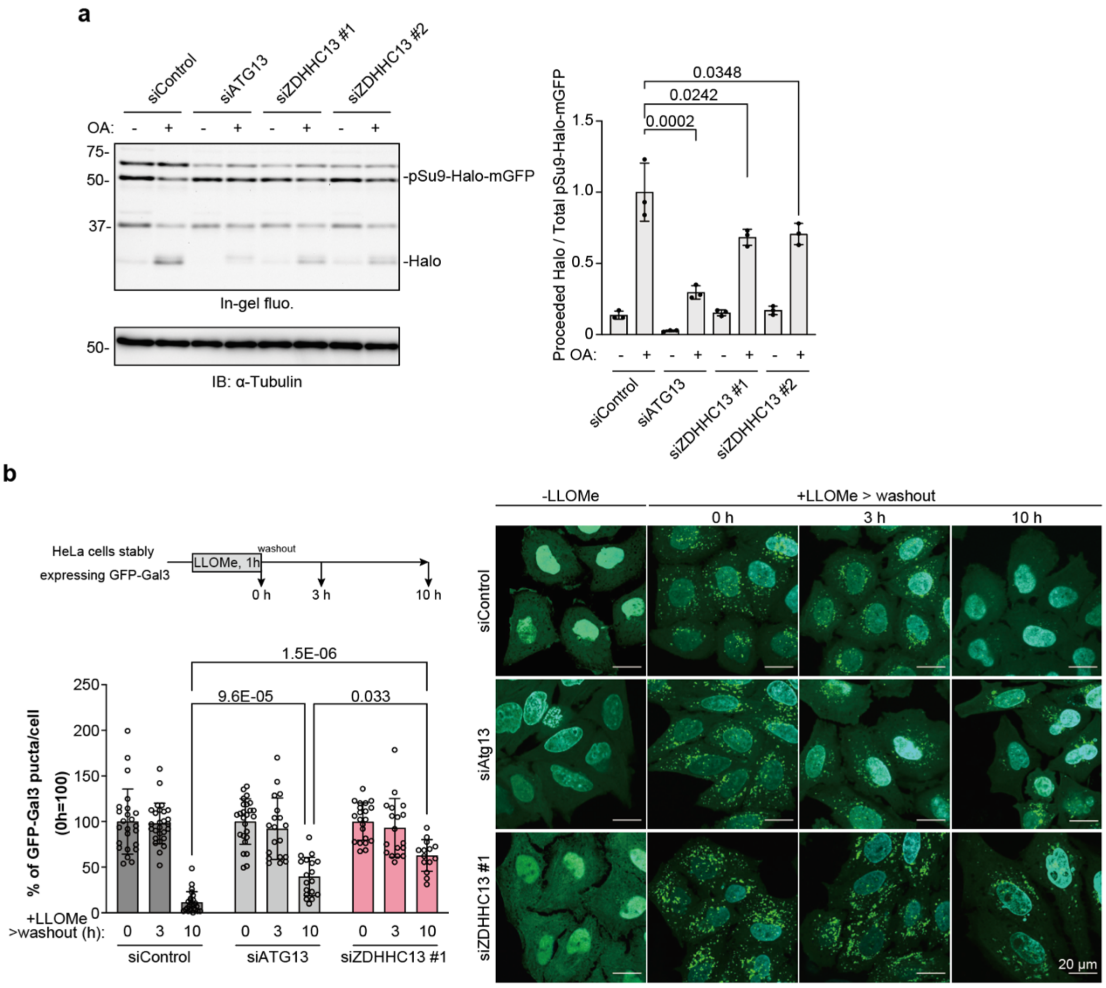

Supplementary Fig. 3

ZDHHC13 knockdown inhibited mitophagy and lysophagy.

a HeLa cells stably expressing pSu9-Halo-mGFP and Myc-Parkin were treated with siRNAs for two days. The cells were pulse-labeled for 20 min with TMR-conjugated ligands and incubated in growth medium containing oligomycin and antimycin for 6 h. Representative images from in-gel fluorescence and Immunoblotting are show in left. The graph is represented as mean ± SD from three experiments. Significance was calculated by one way-ANOVA. **b** HeLa cells stably expressing GFP-galectin-3 (Gal3) were treated with or without 1,000 μM LLOMe for 1 h, and the LLOMe was washed out; then, the cells were incubated for an additional 0, 3, or 10 h. Representative images from microscopy are show in right. Nuclei were stained with DAPI. GFP-Gal3 and DAPI signals are shown as green and cyan, respectively. The graph is represented as

mean ± SD from more than 23 cells. Significance was calculated by one way-ANOVA. The experiment was independently repeated twice and showed similar results.

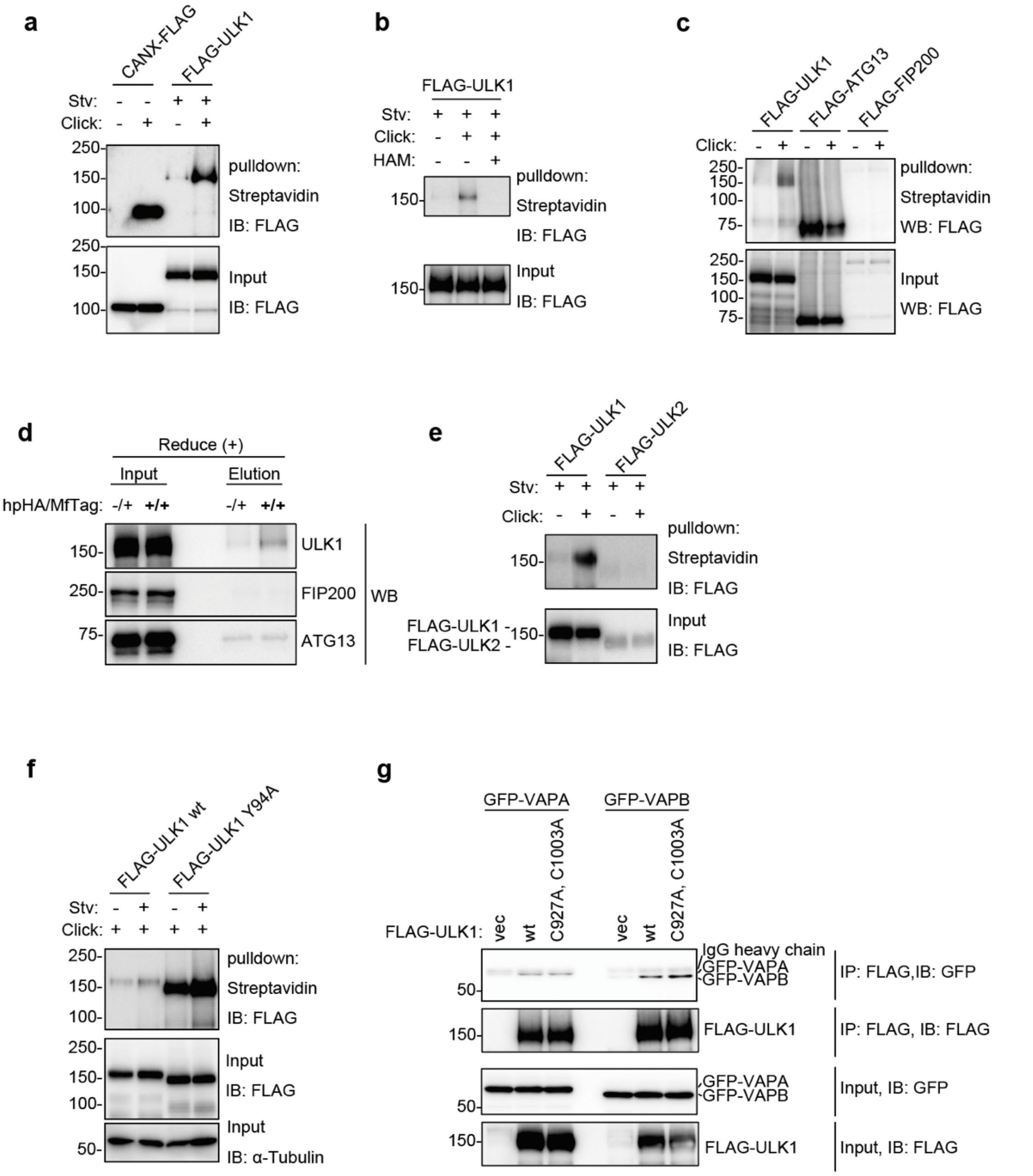

Supplementary Fig. 4

The palmitoylation of ULK1 is independent on the interaction with VAPs.

a In The palmitoylation assay, precipitated bands of Calnexin (CANX) and ULK1 were detected in click reaction samples. FLAG-ULK1 or CANX-FLAG was transiently expressed in HeLa cells. The cells were incubated in growth medium (Stv-) or EBSS (Stv+) for 2 h before lysis. Click- sample was not treated with 17-ODYA. Input lysate

and eluates were analyzed by immunoblotting with anti-FLAG antibody. Input samples and pulldown samples were loaded at the ratios; Input : Pulldown=1 : 60 (CANX) and 1 : 20 (ULK1) during SDS-PAGE. **b** Effect of hydroxylamine (HAM, NH_2OH) treatment on the click reaction. HeLa cells transiently expressing FLAG-ULK1 were prepared as shown in a. For a HAM treatment sample, final 2.5% of hydroxylamine was added in reaction tube as described in Methods. Protein samples were analyzed by immunoblotting. **c** FLAG-ULK1, FALG-ATG13 or FLAG-FIP200 were transiently expressed and subjected to the palmitoylation assay. **d** Palmitoylation assay using S-palmitoylation detection kit was performed as described in Methods. hpHA- is used as a cleavage control of thioester bond. **e** HeLa cells transiently expressed with FLAG-ULK1 or FLAG-ULK2 were subjected to the palmitoylation assay. **f** Palmitoylation of ULK1 Y94A mutant. FLAG-ULK1 wt or Y94A mutant was transiently expressed in HeLa cells. The cells were incubated in growth medium (Stv-) or EBSS (Stv+) for 2 hours before lysis. Cell lysates were treated as described in Methods. Input lysate and eluates were analyzed by immunoblotting with anti-FLAG antibody. **g** Interaction of ULK1 (C927A, C1003A) mutant with VAPA or VAPB. HeLa cells stably expressing GFP-VAPA or VAPB were transfected with vector, FLAG-tagged ULK1 or ULK1 mutants. The cells were lysed after 48 hours and subjected to immunoprecipitation using anti-FLAG M2 agarose beads. Input lysate and eluates were analyzed by immunoblotting with anti-FLAG antibody or anti-GFP antibody. For immunoblotting in Supplementary Fig. 4, the experiment was performed twice and representative images are shown.

Table S1. Materials

REAGENT or RESOURCE	SOURCE	IDENTIFIER
Primary Antibodies		
goat polyclonal anti-ZDHHC13	abcam	ab28759
Rabbit polyclonal anti-ATG13	Sigma-Aldrich	SAB4200100
Rabbit polyclonal anti-LC3	MBL	PM036
Rabbit monoclonal anti-ULK1 (D8H5)	Cell Signaling Technology	8054
Rabbit polyclonal anti-phospho-ULK1 (Ser 757)	Cell Signaling Technology	6888
Rabbit polyclonal anti-p70 S6 Kinase	Cell Signaling Technology	9202
Rabbit polyclonal anti-phopho-p70 S6 Kinase (Thr389)	Cell Signaling Technology	9205
Rabbit monoclonal anti-mTOR (7C10)	Cell Signaling Technology	2983
Rabbit polyclonal anti-TFEB	Cell Signaling Technology	4240
Rabbit monoclonal anti-ATG9A (D4O9D)	Cell Signaling Technology	13509
Rabbit monoclonal anti-Phospho-ATG14L (Ser29) (M4B8M)	Cell Signaling Technology	92340
Mouse monoclonal anti-ATG14L	MBL	M184-3
Mouse monoclonal anti-α-Tubulin	Sigma-Aldrich	T5168
Rabbit polyclonal anti-calnexin	Cell Signaling Technology	2433
Mouse monoclonal anti-WIPI2 (2A2)	abcam	ab105459
Rabbit polyclonal anti-FIP200	Proteintech	17250-1-AP
Mouse monoclonal anti-GM130	BD Transduction Laboratories	610822
Rabbit polyclonal anti-GFP	MBL	598
Mouse monoclonal anti-FLAG (M2)	Sigma-Aldrich	F3165
Rabbit polyclonal anti-Myc-tag	MBL	562
Rabbit polyclonal anti-HA tag	Thermo Fisher Scientific	PA1-985
Secondary Antibodies		
Goat-anti-Rabbit(H+L)-HRP	Jackson Immuno Research Laboratories, Inc.	111-035-003
Goat-anti-Mouse(H+L)-HRP	Jackson Immuno Research Laboratories, Inc.	115-035-003
Donkey-anti-goat (H+L)-HRP	Jackson Immuno Research Laboratories, Inc.	705-035-003
Alexa Fluor 488 donkey anti-rabbit IgG	Thermo Fisher Scientific	A-21206

Alexa Fluor 488 donkey anti-mouse IgG	Thermo Fisher Scientific	A-21202
Alexa Fluor 488 donkey anti-mouse IgG2a	Thermo Fisher Scientific	A-21131
Alexa Fluor 568 donkey anti-rabbit IgG	Thermo Fisher Scientific	A-10042
Alexa Fluor 568 donkey anti-mouse IgG	Thermo Fisher Scientific	A-10037
Alexa Fluor 568 donkey anti-mouse IgG1	Thermo Fisher Scientific	A-21124

**Reagents and Chemicals**		
Dulbecco's modified Eagle's medium (DMEM)	Sigma-Aldrich	D6429
Fetal bovine serum (FBS)	Sigma-Aldrich	F7524
Penicillin-Streptomycin	Sigma-Aldrich	P4333
L-Glutamine Solution	Sigma-Aldrich	G7513
Trypsin/EDTA	Sigma-Aldrich	T4174
Earle’s Balanced Salts (EBSS)	Sigma-Aldrich	E2888
Bafilomycin A1	Sigma-Aldrich	B1793
2-bromopalmitate (2-BP)	FUJIFILM Wako Pure Chemical Corporation	320-76562
4%-Paraformaldehyde Phosphate Buffer Solution	Nakarai Tesque	09154-85
Lipofectamine RNAiMAX Transfection Reagent	Thermo Fisher Scientific	13778150
TransIT-LT1 reagent	Mirus	MIR2300
polyethylenimine (PEI MAX)	Polysciences Inc.	24765
Opti-MEM reduced serum Medium	Thermo Fisher Scientific	31985070
polybrene	Sigma Aldrich	H9268
Mounting Medium	VECTASHIELD	H-1000
Mounting Medium with DAPI	VECTASHIELD	H-1200
cOmplete, EDTA-free Protease Inhibitor Cocktail	Roche	4693132001
PMSF	Nakarai Tesque	06297-31
Immobilon Forte Western HRP substrate	Merck	WBLUF0500
17-ODYA	Cayman	90270
CuSO4	Sigma Aldrich	451657
TCEP	Sigma Aldrich	C4707
TBTA	Sigma Aldrich	678937
Azide-PEG3-biotin conjugate	Sigma Aldrich	762024
Streptavidin-magnetic beads	Thermo Fisher Scientific	88816
anti-FLAG M2 agarose beads	Sigma-Aldrich	A2220
anti-HA magnetic beads	Thermo Fisher Scientific	88836

In-Fusion HD Cloning Plus	TAKARA Bio	638909
RNeasy Plus Mini Kit	Qiagen	74134
Gateway LR Clonase II Enzyme mix	Thermo Fisher Scientific	11791020
GEL/PCR purification mini kit	FAVORGEN	FAGCK 001
NucleoSpin Plasmid EasyPure	TAKARA Bio	U0727C
NucleoBond Xtra Midi	TAKARA Bio	U0410B
tetramethyl rhodamine (TMR)-conjugated ligands	Promega	G8252
oligomycin	Sigma Aldrich	495455
antimycin	Sigma Aldrich	A8674
hydroxylamine (HAM, NH_2OH)	FUJIFILM Wako Pure Chemical Corporation	088-07221
protein S-palmitoylation detection kit (RapidsPALM)	BioDynaics Laboratory Inc.	F017B
Leu-Leu methylester hydrobromide (LLOMe)	Sigma-Aldrich	L7393
Strep-Tactin XT 4 Flow high capacity	iba	2-5030-002
**Cell lines**		
HeLa Kyoto	N/A	N/A
Lenti-X 293T	TAKARA Bio	632180
Plat-E	[1]	(1)
COS7	[2]	N/A

**Plasmids**		
pLP-VSVG	This study	N/A
pMRX-ires-puro	Dr. S. Yamaoka (Tokyo Medical and Dental University)	N/A
pMRX-ires-puro_tfLC3	[3]	N/A
pMRX-ires-puro_EGFP-LC3	[4]	
pMRX-ires-puro_EGFP-Atg5	[4]	N/A
pMRX-ires-puro_ULK1-EGFP	[4]	N/A
pMRX-ires-puro_EGFP-WIPI1	[4]	N/A
pMRX-ires-puro_EGFP-VAPA	This study	N/A

pMRX-ires-puro_EGFP-VAPB	This study	N/A
pMRX-ires-puro_mNG	This study	N/A
pMRX-ires-puro_mNG-ULK1 wt	This study	N/A
pMRX-ires-puro_mNG-ULK1 C927A	This study	N/A
pMRX-ires-puro_mNG-ULK1 C1003A	This study	N/A
pMRX-ires-puro_mNG-ULK1 C927A, C1003A	This study	N/A
pMRX-ires-puro_ZDHHC13-mNG	This study	N/A
pMRX-ires-puro_ZDHHC13-mStrawberry	This study	N/A
pMRX-ires-bla_mScarlet-ULK1 wt	This study	N/A
pMRX-ires-bla_dTomato-ATG5	This study	N/A
pMRX-ires-bla_ATG9A-mCherry	This study	N/A
pMRX-ires-puro-HaloTag7-LC3	Addgene	184899
pMRX-ires-bla-pSu9-Halo-mGFP	Addgene	184905
pMRX-ires-puro-EGFP-Galectin3	[5]	N/A
pMRX-ires-puro_Myc-Parkin	This study	N/A
pMRX-ires-puro-YFP-ATG5	[2]	N/A
pMRX-ires-puro-ATG9A-CFP	This study	N/A
pcDNA3.1+	Thermo Fisher Scientific	V79020
pcDNA3.1+_3xFLAG-ULK1 wt	This study	N/A
pcDNA3.1+_3xFLAG-ULK1 C927A	This study	N/A
pcDNA3.1+_3xFLAG-ULK1 C950A	This study	N/A
pcDNA3.1+_3xFLAG-ULK1 C1003A	This study	N/A
pcDNA3.1+_3xFLAG-ULK1 C1033A, C1036A	This study	N/A
pcDNA3.1+_3xFLAG-ULK1 C927A, C1003A	This study	N/A
pcDNA3.1+_3xFLAG-ULK1 K46I	This study	N/A
pcDNA3.1+_3xFLAG-ULK1 K46N	This study	N/A
pcDNA3.1+_3xFLAG-ULK1 Y94A	This study	N/A
pcDNA3.1+_3xFLAG-ULK2 wt	This study	N/A
pcDNA3.1+_3xFLAG-ATG13	This study	N/A
pcDNA3.1+_3xFLAG-FIP200	This study	N/A
pcDNA3.1+_FLAG-ULK1	This study	N/A
pcDNA3.1+_ULK1-HA	This study	N/A
pcDNA3.1+_Myc-FIP200	This study	N/A
pcDNA3.1+_Myc-ATG13	This study	N/A

pCAG_CANX-FLAG	This study	N/A
pCAG_ZDHHC13 wt-Strep-FLAG	This study	N/A
pCAG_ZDHHC13 wt, siRNA-resistant-Strep-FLAG	This study	N/A
pCAG_ZDHHC13 (D453A, Q454A), siRNA-resistant-Strep-FLAG	This study	N/A
pCAG_Myc-FIP200	This study	N/A
pCAG_ULK1-HA	This study	N/A
pCAG_Myc-ATG13	This study	N/A
pLJC5_ATG9A-3xHA	This study	N/A
pRS316_YFP-Atg1 wt	This study	N/A
pRS316_YFP-Atg1 C731A, C817A	This study	N/A
lentiCRISPRv2	Addgene	52961
**siRNAs**		
siLucsiferase: 5′-UCGAAGUAUUCCGCGUACGdTdT-3′	This study	N/A
siSNX24: 5'-CGACGACAAGGCUUGGAAAtt-3'	This study	N/A
siZDHHC13 #1: 5′-GGACUUACCUCAAUCAGAUtt-3′	Ambion	s29091
siZDHHC13 #2: 5′-CAAGGUUCUUGGUUGGGUAtt-3′	Ambion	s29092
siAtg13: 5′-GAGUUUGGAUAUACCCUUUtt-3′	This study	N/A
**CRISPR-Cas9_guide RNAs used to generate ZDHHC13 KO cells**		
ZDHHC13 #1: 5-GTCATATTATTACTCCAGCA-3	This study	N/A
ZDHHC13 #2: 5-GAGATGAGATATGCTATAAT-3	This study	N/A
ZDHHC13 #3: 5-TTGTGAGCTGATAACATGAG-3	This study	N/A
ZDHHC13 #4: 5-GTCATATTATTACTCCAGCA-3	This study	N/A
ZDHHC13 #5: 5-GAGGTGGCTGCAGAAATGCG-3	This study	N/A
FIP200: 5-AGAGTGTGTACCTACAGTGC-3	This study	N/A
**Cloning primers**		
ZDHHC13: 5’-aaaaggatccgccaccATGGAGGGGCCGGGGCTGG-3’ and 5’-aaaatctcgagtgTACTGAGCGAAGAACCTTCTCCCTGG-3’	This study	N/A
VAPA: 5’-tcagtcgactggatccggATGGCGTCCGCCTCAGGG-3’ and 5’-gtctagatatctcgagCTACAAGATGAATTTCCC-3’	This study	N/A

VAPB: 5’-tcagtcgactggatccggATGGCGAAGGTGGAGCAG-3’ and 5’-gctgggtctagatatcCTACAAGGCAATCTTCCC-3’	This study	N/A

**Software and Algorithms**		
Fiji (ImageJ version: 2.0.0-rc-69/1.52n)	N/A	https://fiji.sc
Excel version: 16.58	Microsoft	N/A
Prism9 (version: 9.3.1)	GraphPad	https://www.graphpad.com
CellProfiler	Broad Institute	https://cellprofiler.org

Cell line	Base cell line	resistance	reference
HeLa_tfLC3	HeLa	puromycin	[3]
HeLa_EGFP-LC3	HeLa	puromycin	[4]
HeLa_EGFP-ATG5	HeLa	puromycin	[4]
HeLa_EGFP-WIPI1	HeLa	puromycin	[4]
HeLa_EGFP-VAPA	HeLa	puromycin	This study
HeLa_EGFP-VAPB	HeLa	puromycin	This study
HeLa_ZDHHC13-EGFP	HeLa	puromycin	This study
HeLa_ZDHHC13-mNG	HeLa	puromycin	This study
HeLa_mNG-ULK1 wt	HeLa	puromycin	This study
HeLa_mNG-ULK1 C927A	HeLa	puromycin	This study
HeLa_mNG-ULK1 C1003A	HeLa	puromycin	This study
HeLa_mNG-ULK1 C927A, C1003A	HeLa	puromycin	This study
HeLa_ZDHHC13-mNG_mScarlet-ULK1	HeLa	puromycin, blasticidin	This study
HeLa_ZDHHC13-mNG_tdTomato-ATG5	HeLa	puromycin, blasticidin	This study
HeLa_ZDHHC13-mNG_ATG9A-mCherry	HeLa	puromycin, blasticidin	This study
HeLa_Halo-LC3	HeLa	puromycin	This study

HeLa_pSu9-Halo-mGFP_Myc-Parkin	HeLa	puromycin, blasticidin	This study

Supplementary Movie 1: Live-cell imaging for ZDHHC13-mNG and ATG9A-mCherry

HeLa cells stably expressing ZDHHC13-mNG and ATG9A-mCherry were plated in a glass-bottom dish one day before imaging. The cells were cultured in EBSS for 1 hour, and images was acquired 360 times at 0.35 second interval by Leica Mica. Green and magenta signals indicate ZDHHC13-mNG and ATG9A-mCherry, respectively.

Supplementary References

1. Morita, S., Kojima, T. & Kitamura, T. Plat-E: an efficient and stable system for transient packaging of retroviruses. *Gene Ther* **7**, 1063-1066 (2000).
2. Hamasaki, M. *et al.* Autophagosomes form at ER-mitochondria contact sites. *Nature* **495**, 389-393 (2013).
3. Kimura, S., Noda, T. & Yoshimori, T. Dissection of the autophagosome maturation process by a novel reporter protein, tandem fluorescent-tagged LC3. *Autophagy* **3**, 452-460 (2007).
4. Kageyama, S. *et al.* The LC3 recruitment mechanism is separate from Atg9L1-dependent membrane formation in the autophagic response against Salmonella. *Mol Biol Cell* **22**, 2290-2300 (2011).
5. Fujita, N. *et al.* Recruitment of the autophagic machinery to endosomes during infection is mediated by ubiquitin. *J Cell Biol* **203**, 115-128 (2013).
